# Clinical and genetic characterization of hereditary breast cancer in a Chinese population

**DOI:** 10.1186/s13053-017-0079-4

**Published:** 2017-10-30

**Authors:** Wenjing Jian, Kang Shao, Qi Qin, Xiaohong Wang, Shufen Song, Xianming Wang

**Affiliations:** 1 Department of Breast and Thyroid Surgery, Shenzhen Second people’s Hospital, Shenzhen, 518035 China; 2 Department of Breast and Thyroid Surgery, The Third Affiliated Hospital of Sun Yat-Sen University, Guangzhou, 510630 China; 3 BGI-Shenzhen, Shenzhen, 518083 China

**Keywords:** Hereditary breast cancer, Causative variant, Gene panel, NGS

## Abstract

**Background:**

Breast cancer develops as a result of multiple gene mutations in combination with environmental risk factors. Causative variants in genes such as BRCA1 and/or BRCA2 have been shown to account for hereditary nature of certain breast cancers. However,other genes, such as ATM, PALB2, BRIP1, CHEK, BARD1, while lower in frequency, may also increase breast cancer risk. There are few studies examining the role of these causative variants. Our study aimed to examine the clinical and genetic characterization of hereditary breast cancer in a Chinese population.

**Methods:**

We tested a panel of 27 genes implicated in breast cancer risk in 240 participants using Next-Generation Sequencing. The prevalence of genetic causative variants was determined and the association between causative variants and clinico-pathological characteristics was analyzed.

**Results:**

Causative variant rate was 19.2% in the breast cancer (case) group and 12.5% in the high-risk group. In the case group 2.5% of patients carried BRCA1 causative variant, 7.5% BRCA2 variants, 1.7% patients had MUTYH, CHEK or PALB2 variants, and 0.8% patients carried ATM, BARD1, NBN, RAD51C or TP53 variants. In the high-risk group 5.8% women carried MUTYH causative variants, 2.5% had causative variants in ATM, 1.7% patients had variants in BRCA2 and 0.8% in BARD1, BRIP1 or CDH1. There was no significant difference in the presence of causative variants among clinical stages of breast cancer, tumor size and lymph nodes status. However, eight of the 12 BRCA1/2 causative variants were found in the TNBC group.

**Conclusions:**

We found increased genetic causative variants in the familial breast cancer group and in high-risk women with a family history of breast cancer. However, the variant MUTYH c.892-2A > G may not be directly associated with hereditary breast carcinoma.

**Electronic supplementary material:**

The online version of this article (10.1186/s13053-017-0079-4) contains supplementary material, which is available to authorized users.

## Background

Breast cancer is a common malignancy among women, with an estimated annual rate of incidence increasing by 2–3% in China, especially in metropolitan areas [1]. It is known that while the majority of breast cancers are sporadic in origin, an appreciable fraction result from inherited causative variants [2, 3] . Cancer is caused by the cumulative effects of mutations in multiple genes, in combination with environmental factors. It has been suggested that reproductive and hormonal factors, such as nulliparity, increased age at first live birth, and limited breast feeding are associated with a modest increase in the risk of breast cancer in Western countries [4, 5]. Breast cancer susceptibility genes BRCA1 and BRCA2 causative variant account for only 10–20% of breast cancers with a known family history [6]. The prevalence of hereditary breast cancers is approximately 11.8% in China [7], suggesting that other genes may play an important role in increasing the susceptibility to breast cancer, albeit at a markedly lower frequency and penetrance. For example, women with inherited causative variant in the Fanconi anemia genes BRIP1 and PALB2 have a 20–50% lifetime risk of breast cancer [8, 9]. Multiple studies have also demonstrated that genes such as ATM [10–12] and CHEK2 [13–16] are associated with increased breast cancer risk. In addition, inherited causative variants in TP53, PTEN, STK11, and CDH1 are associated with a moderate to very high-risk of developing breast cancer [17–20].

Although studies have demonstrated the clinical benefit of multiple-gene sequencing for the assessment of patients with high-risk hereditary cancer [21, 22], little information is currently available regarding the value of multiple-gene sequencing for the assessment of the risk of hereditary breast cancer in China. The goal of this study was to identify the variant spectrum for the clinical and genetic characterization of familial breast cancer in a Chinese population. Twenty-seven breast cancer susceptibility genes (Additional file 1: Table S1), selected through a database (HGMD: Human Gene Mutation Database, NCBI ClinVar database) and published research articles, were tested by Next-Generation Sequencing (NGS).

## Methods

### Patients and samples

In total, 240 participants, including 120 patients with breast cancer and 120 high-risk women with first- or second-degree relative(s) suffering from breast cancer were recruited from Shenzhen Second People’s Hospital of China during a two year period (2014–2016). The rate of susceptibility gene causative variants in East Asian population in 1000 Genomes database was used as a control. The clinical breast cancer diagnosis and classification criteria were in accordance with the World Health Organization criteria. Written informed consent was obtained from patients and healthy high-risk women. The study was approved by a local ethics committee. Two hundred and forty peripheral blood samples were collected and referred for genetic testing to the BGI research Department (Shenzhen, China).

### Sample treatment, next-generation sequencing and variants calling

DNA was extracted from participants’ peripheral blood samples using a Qiagen DNA blood mini kit (Qiagen, Hilden, Germany) according to the manufacturer’s recommendations. Qubit Fluorometer (Life Technologies) and agarose gel electrophoresis were used to determine DNA concentration and purity. Genomic DNAs were randomly fragmented to 200-300 bp by Covaris E210 (Massachusetts, USA) and treated as follows: end-repair, A-tailing and adapter ligation, and PCR amplification. PCR products were captured by the same BGI chip in the Blackbird platform. Their frequency was determined by quantitative PCR, and the segments were pooled for sequencing on the Hiseq 2500 (Illumina) according to the manufacturer’s protocols. Over 0.6 GB data was generated per sample with approximately 200X depth and over 99% coverage of the target region. Variants were detected using Small Variant Assembler Methods (http://www.completegenomics.com/documents/Small_Variant_Assembler_Methods.pdf) which is available on the official website of Complete Genomics. Then, variants were filtered according to their read support, assemble quality and reference allele repeat status. Sequences generated by high-throughput sequencing platforms were filtered by SOAPnuke1.5.0 with standard augmentation, and then assembled by BWA 0.7.12 using MEM. Sam Tools 1.2 was used to convert file format into BAM. Base quality was recalibrated by GATK 3.4. Duplications were removed by Picard Mark Duplicates 1.138. Local realignment of reads around insertion/deletion was performed and variants were called by insertion/deletion Realigner and Haplotype Caller in GATK 3.4. Variants were further filtered by quality depth, strand bias, mapping quality and reads position.

### Variant classification

In accordance with the American College of Medical Genetics (ACMG) recommendations for the interpretation of sequence, variants were classified into pathogenic, likely pathogenic, variant of uncertain significance (VUS), likely benign, and benign variant. Variants were classified as pathogenic if they conferred truncations, or initiation codons, affected splicing or if they have been reported in the central mutation database (HGMD, ClinVar), or in published literature, and demonstrated to be causative of the disorder in a particular disease with no conflicting results. Variants were classified as VUS if they fulfilled the following three criteria at the same time: 1) missense, non-frame shift or intronic (exon-intron boundaries ±10 bp) variants, and 2) allele frequency in the 1000 Genomes Study and 101 BGI normal Chinese genomes study are both less than 0.03, and 3) variants were not uniformly identified as benign/likely benign in ClinVar. The rest of variants were identified as benign. In addition, every pathogenic variant detected by next-generation sequencing was confirmed by conventional PCR-Sanger sequencing. Twenty-seven genes examined in this study (Additional file 1: Table S1) were selected through database or published articles about known mutations in hereditary breast cancer.

### Statistics

Statistical tests were carried out using SPSS 20.0 (IBM, Armonk, NY), applying chi-square or Fisher’s exact tests when required to analyze categorical data. A *p* values less than 0.05 was considered as statistically significant.

## Results

### Characteristics of the study population

We recruited for this trial 120 patients diagnosed with breast cancer and 120 high-risk women who had first-degree relatives affected by breast cancer. Table 1 summarizes the risk factor data of the study population reflecting the epidemiology of breast cancer. The median age at blood sample collection was 46 years (range from 25 to 81 years) in the breast cancer group and the median age was 37 years in the high-risk group. There were no statistically significant differences in body mass index (BMI), age at menarche, and breast-feeding history. However, there were statistically significant differences between the two groups in parity and abortion rates. In this study 77.5% of patients had no history of childbearing and 41.7% of patients had a history of abortion, which may confer a high-risk of breast cancer in Chinese individuals.

**Table 1:** Epidemiological characteristics of the study participants

Variable	No (BC) (%)(*n* = 120)	No (high-risk group) (%)(n = 120)	*P*-Value
The median age at sample collection (Range)	46(25–81)	37(18–77)	
BMI(kg/m^2^)			0.095
< 25	79(65.8)	93(77.5)	
≥25	24(20.0)	13(10.8)	
Unknown	17(14.2)	14(11.7)	
Age at menarche(in years)			0.815
< 13	21(17.5)	24(20.0)	
≥13	76(63.3)	76(63.3)	
Unknown	23(19.2)	20(16.7)	
Parity			0.005
Nulliparous	93(77.5)	80(66.7)	
Parous	7(5.8)	24(20.0)	
Unknown	20(16.7)	16(13.3)	
Breast-feeding history			0.094
Yes	65(54.2)	50(41.7)	
No	18(15.0)	29(24.2)	
Unknown	37(30.8)	41(34.2)	
Abortion			0.017
Yes	50(41.7)	33(27.5)	
No	50(41.7)	72(60.0)	
Unknown	20(16.6)	15(12.5)	

### Prevalence of panel-gene causative variants in the two groups

In order to explore the presence of predisposing genetic factors for the development of breast cancer, all participants were subjected to a multiple-gene panel sequencing and variant analysis. The presence of 27 causative variants (Additional file 1: Table S1) associated with an increased susceptibility to breast cancer was tested in this panel using NGS. As showed in Table 2, the ratio of variants in the breast cancer group was 19.2% (23/120) and 12.5% (15/120) in the high-risk group. Twelve predisposing causative variants in 27 panel-genes were identified in this study. Three (2.5%) in BRCA1, nine (7.5%) in BRCA2, two (1.7%) each in MUTYH, CHEK and PALB2, one (0.8%) each in ATM, BARD1, NBN, RAD51C, TP53 were identified in the breast cancer group, while seven (5.8%) in MUTYH, three (2.5%) in ATM, two (1.7%) in BRCA2, one (0.8%) each in BARD1, BRIP1 and CDH1 were identified in the high-risk group. There were no causative variants found in other genes examined.

**Table 2:** Distribution of multiple-gene variants in two groups of 240 participants

Variable	No (BC) (%)(*n* = 120)	No (high-risk group) (%)(*n* = 120)	*P*-Value
BRCA1	3(2.5)	0(0.0)	0.247
BRCA2	9(7.5)	2(1.7)	0.031
ATM	1(0.8)	3(2.5)	0.622
MUTYH	2(1.7)	7(5.8)	0.171
BARD1	1(0.8)	1(0.8)	1.0
BRIP1	0(0.0)	1(0.8)	1.0
CHEK2	2(1.7)	0(0.0)	0.498
NBN	1(0.8)	0(0.0)	1.0
PALB2	2(1.7)	0(0.0)	0.498
RAD51C	1(0.8)	0(0.0)	1.0
TP53	1(0.8)	0(0.0)	1.0
No causative variants	97(80.9)	106(88.4)	0.157

All germline changes revealed by panel sequencing were termed germ line causative variants by the 5-tier rating system. We have excluded “likely benign”, “benign” variants and VUS in the paper, and have listed “pathogenic”, “likely pathogenic,” changes in Tables 3 and 4. Detailed information regarding causative variants in the breast cancer group and the high-risk group (women with a family history of breast cancer) is listed in Tables 3 and in Table 4. Genetic causative variants identified were heterozygous mutations, and most were frameshift deletions. We did not include healthy women with no known history of familial breast cancer in our study, however frequencies of gene causative variants that we identified were examined in healthy population by surveying available databases: http://www.internationalgenome.org/ and http://www.ncbi.nlm.nih.gov/projects/SNP/. We found that the frequencies of these variants were zero in East Asian population in 1000G_ALL (the frequency of this causative variants in all populations of the human international genome). However, we detected MUTYH gene variants (Intron10, c.892-2A > G) at a rate of 2.77% https://www.ncbi.nlm.nih.gov/projects/SNP/snp_ref.cgi?rs=77542170 in East Asian healthy individuals.

**Table 3:** Causative variants identified in the high-risk healthy people

NO.	Year	Gene	Function area	Nucleotide change	AA change	Hom/Het	1000G_ALL	Variant	Annotation	ACMG evidence
SZ010	52	ATM	CDS30	c.4630_4633delTACT	p.Y1544*fsX1	Het	0	frameshift deletion	likely pathogenic	PVS1, PM2
SZ011	57	ATM	CDS30	c.4630_4633delTACT	p.Y1544*fsX1	Het	0	frameshift deletion	likely pathogenic	PVS1, PM2
SZ012	42	BRIP1	CDS9	c.1400delT	p.Ile467AsnfsX9	Het	0	frameshift deletion	likely pathogenic	PVS1, PM2
15B0028764	38	ATM	Exon38	c.5780delT	p.I1927IfsX10	Het	0	frameshift deletion	likely pathogenic	PVS1, PM2
15B0029343	33	BRCA2	CDS21	c.8946_8947delAG	p.K2982KfsX35	Het	0	frameshift deletion	likely pathogenic	PVS1, PM2
15B0027543	28	MUTYH	Intron10	c.892-2A > G	–	Het	0.0277	splicing	likely pathogenic	PVS1, PP5
15B0029366	33	BRCA2	CDS10	c.5344_5345insA	p.Q1782QfsX5	Het	0	frameshift deletion	likely pathogenic	PVS1, PM2
15B0029289	30	BARD1	CDS9	c.1822_1823insT	p.V608VfsX5	Het	0	frameshift deletion	likely pathogenic	PVS1, PM2
15B0027981	61	MUTYH	Intron10	c.892-2A > G	–	Het	0.0277	splicing	likely pathogenic	PVS1, PP5
15B0027558	39	MUTYH	CDS10	c.757C > T	p.Q253X	Het	0	nonsense mutation	pathogenic	PVS1,PM2, PP5
15B0027540	30	MUTYH	Intron10	c.892-2A > G	–	Het	0.0277	splicing	likely pathogenic	PVS1, PP5
15B0027538	35	MUTYH	Intron10	c.892-2A > G	–	Het	0.0277	splicing	likely pathogenic	PVS1, PP5
15B0027537	37	MUTYH	Intron10	c.892-2A > G	–	Het	0.0277	splicing	likely pathogenic	PVS1, PP5
15B0027970	18	MUTYH	Intron12	c.1144 + 2 T > C	–	Het	0	splicing	likely pathogenic	PVS1, PM2

**Table 4:** Causative variants identified in patients with BC

NO.	Year with drawn	Year with affected BC	Gene	Function area	Nucleotide change	AA change	Hom/Het	1000G_ALL	Variant	Annotation	ACMG evidence
SZ007	60	55	RAD51C	CDS4	c.577C > T	p.R193X	Het	0	nonsense mutation	pathogenic	PVS1,PM2, PP5
SZ009	43	39	ATM	CDS30	c.4630_4633delTACT	p.Y1544*fsX1	Het	0	frameshift deletion	likely pathogenic	PVS1, PM2
15B0028780	66	64	BRCA2	intron9	c.793 + 1G > C	–	Het	0	splicing	likely pathogenic	PVS1, PM2
15B0028776	42	41	BRCA2	intron9	c.793 + 1G > C	–	Het	0	splicing	likely pathogenic	PVS1, PM2
15B0029034	38	37	TP53	CDS6	c.733G > A	p.G245S	Het	0	missense mutation	pathogenic	PVS1,PM2, PP5
15B0029040	40	38	BRCA2	intron15	c.7617 + 1G > A		Het	0	splicing	pathogenic	PVS1,PM2, PP5
15B0029035	60	54	BRCA2	intron15	c.7617 + 1G > A		Het	0	splicing	pathogenic	PVS1,PM2, PP5
15B0029311	60	50	BRCA2	CDS21	c.8946_8947delAG	p.K2982KfsX35	Het	0	frameshift deletion	likely pathogenic	PVS1, PM2
15B0027630	46	46	BRCA2	CDS22	c.9100C > T	p.Q3034X	Het	0	nonsense mutation	pathogenic	PVS1,PM2, PP5
15B0029313	57	51	MUTYH	Intron10	c.892-2A > G	–	Het	0.0277	splicing	likely pathogenic	PVS1, PP5
15B0029264	42	42	BRCA2	CDS10	c.5344_5345insA	p.Q1782QfsX5	Het	0	frameshift deletion	likely pathogenic	PVS1, PM2
15B0029350	54	54	BARD1	CDS9	c.1822_1823insT	p.V608VfsX5	Het	0	frameshift deletion	likely pathogenic	PVS1, PM2
15B0027557	74	74	MUTYH	CDS10	c.757C > T	p.Q253X	Het	0	nonsense mutation	pathogenic	PVS1,PM2, PP5
15B0027660	38	36	BRCA1	CDS9	c.3770_3771delAG	p.E1257GfsX9	Het	0	frameshift deletion	likely pathogenic	PVS1, PM2
SZ006	38	33	NBN	CDS14	c.2140C > T	p.R714X	Het	0	nonsense mutation	pathogenic	PVS1,PM2, PP5
SZ014	59	58	BRCA2	CDS10	c.4046 delT	p.Il349IfsX25	Het	0	frameshift deletion	likely pathogenic	PVS1, PM2
15B0029261	63	54	PALB2	CDS5	c.2257C > T	p.R753X	Het	0	nonsense mutation	pathogenic	PVS1,PM2, PP5
15B0027569	66	66	PALB2	intron5	c.2515-2A > G		Het	0	splicing	likely pathogenic	PVS1, PM2
15B0027884	34	34	BRCA1	CDS9	c.3436_3439delTGTT	p.C1146LfsX8	Het	0	frameshift deletion	pathogenic	PVS1,PM2, PP5
16B0005787	46	44	BRCA1	CDS9	c.3114delA	p.E1038EfsX10	Het	0	frameshift deletion	likely pathogenic	PVS1, PM2
15B0027669	41	39	BRCA2	CDS9	c.1399A > T	p.K467X	Het	0	nonsense mutation	pathogenic	PVS1,PM2, PP5

### Association between genetic causative variants and clinicopathological characteristics

Gene causative variants prevalence was 69.6% (16/23) in patients with invasive ductal carcinoma (IDC), 4.3% (1/23) patients with ductal carcinoma in situ (DCIS) and 26.1% (6/23) with an unknown histological type (Table 5). There was no significant difference in the presence of variants between clinical stages of breast cancer (Pearson’s Chi-squared test *p* = 0.537). Although some patients were lost to follow-up, our data suggest that similar causative variants were found in patients regardless of tumor size and lymph nodes status.

**Table 5:** Comparison of patients with and without a pathogenic variant

Characteristic	without Variants (*n*,%)	with Variant (*n*,%)	*P* value
Patient number	97	23	
Histology type			0.218
IDC	72 (74.2)	16 (69.6)	
DCIS	12 (12.4)	1 (4.3)	
Other	13 (13.4)	6 (26.1)	
Molecular type			0.001
TNBC	12 (12.4)	10 (43.5)	
Non-TNBC	72 (74.2)	9 (39.1)	
Unknown	13 (13.4)	4 (17.4)	
Tumor size			0.288
< =2 cm	35 (36.1)	6 (26.1)	
> 2 cm	46 (47.4)	10 (43.5)	
Unknown	16 (16.5)	7 (30.4)	
Clinical stage			0.537
0	12 (12.4)	1 (4.3)	
I	10 (10.3)	2 (8.7)	
II	32 (33.0)	10 (43.5)	
III	24 (24.7)	3 (13.0)	
IV	4 (4.1)	2 (8.7)	
Unknown	15 (15.5)	5 (21.7)	
Lymph nodes status			0.086
Negative	30 (30.9)	10 (43.5)	
Positive	41 (42.3)	4 (17.4)	
Unknown	26 (26.8)	9 (39.1)	

When analyzed, based on the molecular subtype of breast cancer, the genetic causative variant ratio was 43.5% in patients with triple negative breast cancer (TNBC), 39.1% in patients with non-TNBC, and 17.4% in patients with undetermined molecular subtype (*p* = 0.001) (Table 5). Eight of the 12 BRCA1/2 causative variants were found in the TNBC group. The other two gene variants in the TNBC group were BARD1 and RAD51.

## Discussion

In this clinical study, we examined 27 genes associated with an increased susceptibility to breast cancer (Tables 2, 3 and 4) in patients with breast cancer and in high-risk participants with a family history of breast cancer. In addition to BRCA1/2, genes with an established role in breast cancer, other predisposing genes such as CHEK and PALB2 were evaluated for a possible association with the risk of breast cancer, although their frequency and penetrance was significantly lower. We found causative variants in 12 of the 27 genes examined in the participants (Table 2).

There appeared to be considerable discrepancies in the causative variant rates of BRCA1 and BRCA2 in breast cancer patients in different areas of China. Song [23] reported that the variant ratio of BRCA1 and BRCA2 in Shanghai was 11.4% and 2.9%, respectively, whereas in our study the variant ratio of BRCA1 and BRCA2 in breast cancer patients were 2.5% and 7.5%, respectively (Table 2). The main reason for lower causative variant rates of BRCA1 and higher variant rates of BRCA2 in our study may be the different detection methods used in the studies. PCR-SSCP analysis, examining only four “hot areas” in BRCA1/2 was used in the Song’s study, while whole exon NGS of BRCAs was used in our study. In addition, geographical differences are likely to contribute to discrepancies between results. The participants in the Song’s study mainly were recruited from Eastern and Northern China, while the subjects in our study were largely from Southern and Central China.

**Fig. 1:**
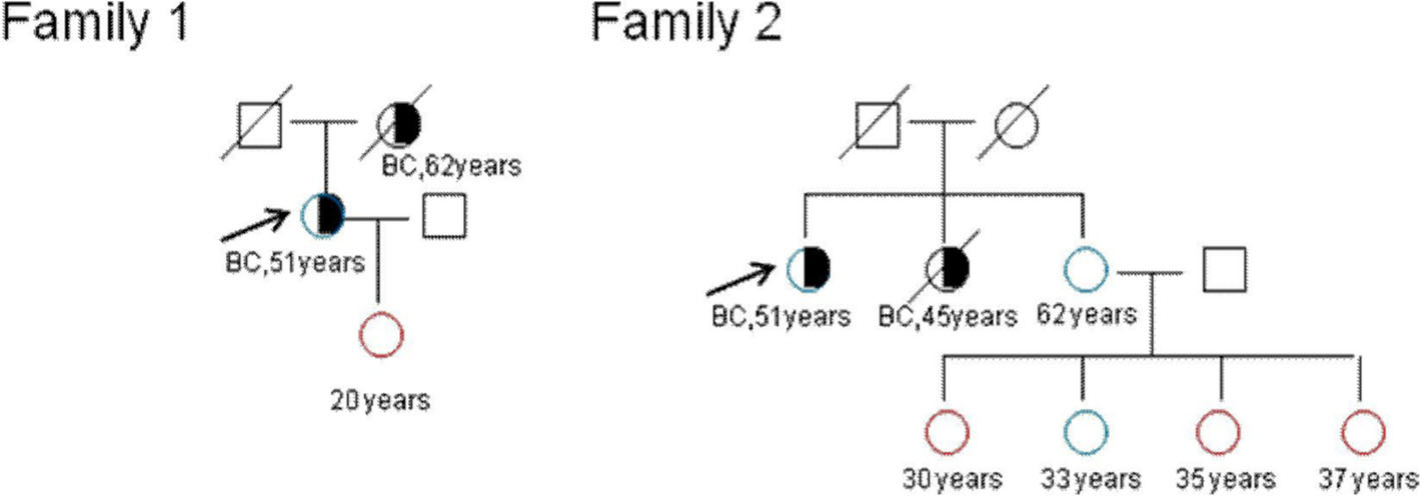
Pedigree maps of two families. stands for MUTYH c.892-2A > G variants. stands for that MUTYH c.892-2A > G variant was not tested. stands for man. stands for woman. and  stand for non-cancerous death.  stands for patient with breast cancer. The black arrow indicates the proband. (BC: Breast cancer)

We found a relatively high variant rate (4.2%, 5/120) of MUTYH c.892-2A > G in the high-risk group, but lower rate (0.8%, 1/120) in the breast cancer group (Table 2). According to the 5-tier rating system in ACMG, this variant is likely pathogenic. However, a correlation between MUTYH variants and breast cancer remains unclear. For example, two other studies suggested a significantly increased breast cancer risk among carriers of the bi-allelic MUTYH variants [24, 25], while other studies showed that germline MUTYH variants are not associated with carcinomas of the breast [26, 27] . In our study, the variant ratio of MUTYH c.892-2A > G in high-risk women with a family history of breast cancer is over 2.77% https://www.ncbi.nlm.nih.gov/projects/SNP/snp_ref.cgi?rs=77542170, the frequency of MUTYH c.892-2A > G in East Asians in 1000G_ALL, but the rate in the breast cancer group is lower. The variant MUTYH c.892-2A > G identified in our study is a heterozygous mutation (Tables 3 and 4). Further, two family pedigrees suggests segregation of this variant (Fig. 1) - the proband did not carry the variant, while their relatives with no BC carried it. Therefore, it is possible that MUTYH c.892-2A > G is a benign variant in the development of BC in East Asians, however we need to enlarge the sample size to confirm this result.

To explore the relationship between gene variants associated with hereditary predisposition and tumor characteristics, we analyzed the association between available pathological and clinical data in breast cancer patients and the presence of gene causative variants. Our results show no statistically significant differences between the presence of gene variants in breast cancer patients and differences in tumor histology, size, clinical stage and lymph node status, however; we found a statistically significant difference in the variant rate in patients with tumors of different molecular type (Table 5). Ten of 22 patients with TNBC were found to harbor gene causative variants. Furthermore, most of TNBC patients (8/10) were found to have BRCA1/2 causative variants. It has been reported that TNBC is common in BRCAs variant carriers [28–31]. Indeed, the incidence of TNBC is around 70% in BRCA1 mutation carriers [32, 33]. Our data are consistent with this observation, however we need to enlarge the sample size to further confirm this association.

As for the clinical significance of the presence of predisposing variants, different advice may be given to specific groups of patients. Patients carrying these pathogenic variants are considered to be at a high-risk in developing tumor recurrence or secondary cancer according to the NCCN guidelines [9, 34]. However, contralateral mastectomy or oophorectomy for these patients is currently not recommended in China, and asymptomatic women carrying pathogenic variants usually prefer not to undergo preventive surgery. In light of this situation, we suggest that patients with a high-risk of developing breast cancer have a comprehensive physical exam every six months, and we advise them to focus on breast self-examination and maintain a healthy life style.

## Conclusion

As the incidence of breast cancer is increasing, it is necessary to carry out more studies to identify susceptibility genes of breast cancer and to establish their frequency. Our results enrich our knowledge of predisposing variants in the population of Southern and Central China, and provide some experimental data for the identification of alternative susceptibility genes, and for the establishment of a clinical model of genetic screening.

However, our study also has some limitations. We did not analyze the relationship between clinicopathological characteristics and gene VUS. More than two hundred VUS were identified in this study, but we have not analyzed them to date. In addition, some patients were lost due to follow-up, which made it difficult to draw conclusions between the association of genetic causative variants and clinicopathological characteristics of patients.

## Additional file

Additional file 1: Table S1. Breast Cancer Susceptibility Gene List [8, 9, 11, 13, 17, 19, 20], [35–47]. (DOC 162 kb)

## Abbreviations

DCIS: Ductal carcinoma in situ
IDC: Invasive ductal carcinoma
NGS: Next-Generation Sequencing
TNBC: Triple negative breast cancer
VUS: Uncertain significance
