# Supplementary material for: Clinical and genetic characterization of hereditary breast cancer in a Chinese population
Source: Hered Cancer Clin Pract. 2017 Oct 30;15:19. doi: 10.1186/s13053-017-0079-4 (PMC5663067; doi:10.1186/s13053-017-0079-4)
Supplement: Additional file 1: Table S1. — Breast Cancer Susceptibility Gene List [8, 9, 11, 13, 17, 19, 20], [35–47]. (DOC 162 kb) [file 13053_2017_79_MOESM1_ESM.doc]

**Table S1. Breast Cancer Susceptibility Gene List**

|  | **No.** | **Gene name** | **Breast Cancer Relative Risk** |  | **References** |
| --- | --- | --- | --- | --- | --- |
| **High-penetrant Genes** | 1 | BRCA1 | 4.0-7.0 |  | Chen, S. |
|  | 2 | BRCA2 | 4.0-7.0 |  | Chen, S. |
|  | 3 | CDH1 | 5.9-7.3 |  | Pharoah, P. D. |
|  | 4 | PTEN | 2.0-5.0 |  | Tan, M. H. |
|  | 5 | STK11 | 4.0-6.7 |  | Apostolou, P. |
|  | 6 | TP53 | 4.3-9.3 |  | Gonzalez, K. D |
| **Moderate-penetrant Genes** | 7 | ATM | 1.5-3.8 |  | Renwick, A |
|  | 8 | BARD1 | unknown |  | Apostolou, P. |
|  | 9 | BRIP1 | 1.2-3.2 |  | Seal, S |
|  | 10 | CHEK2 | 2 fold, 25-37% life time risk |  | Apostolou, P. |
|  | 11 | HMMR | unknown |  | Pujana, M. A. |
|  | 12 | MLH1 | 0.2-2.0 |  | Win, A. K. |
|  | 13 | MLH3 | unknown |  | Conde, J. |
|  | 14 | MSH2 | 1.2-3.7 |  | Win, A. K |
|  | 15 | MSH6 | 0-13 |  | Win, A. K. |
|  | 16 | MUTYH | 1.0-3.4 |  | Rennert, G. |
|  | 17 | NBN | 1.4-6.6 |  | Bogdanova, N. |
|  | 18 | NQO2 | unknown |  | YU, K. D. |
|  | 19 | PALB2 | 1.4-3.9 |  | Rahman,N. |
|  | 20 | PHB | unknown |  | Jupe, E. R. |
|  | 21 | PMS1 | unknown |  | Kobayashi, H |
|  | 22 | PMS2 | unknown |  | Kobayashi, H |
|  | 23 | PPM1D | unknown |  | Ruark, E |
|  | 24 | RAD50 | unknown |  | Apostolou, P. |
|  | 25 | RAD51 | unknown |  | Li, W |
|  | 26 | RAD51C | 1.5-7.8 |  | Meindl, A. |
|  | 27 | XRCC3 | unknown |  | Kuschel, B. |
